# Dietary Indole-3-Carbinol Activates AhR in the Gut, Alters Th17-Microbe Interactions, and Exacerbates Insulitis in NOD Mice

**DOI:** 10.3389/fimmu.2020.606441

**Published:** 2021-01-21

**Authors:** Heather M. Kahalehili, Nolan K. Newman, Jamie M. Pennington, Siva K. Kolluri, Nancy I. Kerkvliet, Natalia Shulzhenko, Andrey Morgun, Allison K. Ehrlich

**Affiliations:** ^1^Department of Environmental Toxicology, University of California, Davis, CA, United States; ^2^College of Pharmacy, Oregon State University, Corvallis, OR, United States; ^3^Department of Environmental and Molecular Toxicology, Oregon State University, Corvallis, OR, United States; ^4^Department of Biomedical Sciences, Oregon State University, Corvallis, OR, United States

**Keywords:** indole-3-carbinol (I3C), type 1 diabetes, aryl hydrocarbon receptor (AhR), Th17 cells (Th17), microbiome

## Abstract

The diet represents one environmental risk factor controlling the progression of type 1 diabetes (T1D) in genetically susceptible individuals. Consequently, understanding which specific nutritional components promote or prevent the development of disease could be used to make dietary recommendations in prediabetic individuals. In the current study, we hypothesized that the immunoregulatory phytochemcial, indole-3-carbinol (I3C) which is found in cruciferous vegetables, will regulate the progression of T1D in nonobese diabetic (NOD) mice. During digestion, I3C is metabolized into ligands for the aryl hydrocarbon receptor (AhR), a transcription factor that when systemically activated prevents T1D. In NOD mice, an I3C-supplemented diet led to strong AhR activation in the small intestine but minimal systemic AhR activity. In the absence of this systemic response, the dietary intervention led to exacerbated insulitis. Consistent with the compartmentalization of AhR activation, dietary I3C did not alter T helper cell differentiation in the spleen or pancreatic draining lymph nodes. Instead, dietary I3C increased the percentage of CD4^+^RORγt^+^Foxp3^-^ (Th17 cells) in the lamina propria, intraepithelial layer, and Peyer’s patches of the small intestine. The immune modulation in the gut was accompanied by alterations to the intestinal microbiome, with changes in bacterial communities observed within one week of I3C supplementation. A transkingdom network was generated to predict host-microbe interactions that were influenced by dietary I3C. Within the phylum Firmicutes, several genera (*Intestinimonas*, *Ruminiclostridium* 9, and unclassified Lachnospiraceae) were negatively regulated by I3C. Using AhR knockout mice, we validated that *Intestinimonas* is negatively regulated by AhR. I3C-mediated microbial dysbiosis was linked to increases in CD25^high^ Th17 cells. Collectively, these data demonstrate that site of AhR activation and subsequent interactions with the host microbiome are important considerations in developing AhR-targeted interventions for T1D.

## Introduction

Type 1 diabetes (T1D) is characterized by uncontrolled hyperglycemia resulting from autoimmune-mediated destruction of insulin-producing beta cells. Unlike other immune-mediated diseases that can be treated by combatting the underlying inflammatory response, T1D is generally diagnosed after the majority of beta cells have been destroyed. Early interventions targeting risk factors that influence T1D progression could negate the need for life-long insulin replacement (1). In genetically susceptible individuals, the diet is among the external factors known to influence the rate of T1D development (2, 3). The influence of diet on T1D is thought to reflect modulation of mucosal immune responses, intestinal permeability, and microbiome diversity (4–7).

One dietary component that has been shown to modulate autoimmune disease development is ligands that activate the aryl hydrocarbon receptor (AhR) (8). AhR modulates many aspects of the autoimmune response and is also becoming known as a major regulator of gut homeostasis (9). Several studies have shown that AhR activation by high affinity ligands suppresses immune-mediated diseases, including T1D (10–14). However, the immunological outcome of dietary AhR ligand intake could be unpredictable due to the heterogeneity of ligands, their AhR affinity, and amount consumed. While a diet rich in AhR ligands would be predicted to mimic the studies showing the therapeutic benefit of high affinity ligands in preventing the progression of T1D, it is possible that consumption of a diet low in AhR ligands could contribute to T1D progression. This concern arises based on our recent studies demonstrating that a low level of AhR activation can lead to the differentiation of Th17 cells instead of Tregs (15), which could promote, rather than prevent T1D progression (16–19).

Indole-3-carbinol (I3C), is a dietary AhR ligand precursor formed as a hydrolysis product of glucobrassicin, a compound found in cruciferous vegetables such as broccoli, kale, and brussel sprouts. I3C is further broken down by the acidic environment in the stomach into dimers, trimers and tetramers, some of which have high affinity for the AhR (20). Intake of I3C has been estimated at 0.1–1.6 mg/kg (21); I3C intake would be lower in individuals who avoid cruciferous vegetables, and higher in those who consume a cruciferous rich diet or who take widely available I3C supplements. The impact of dietary I3C on the inflammatory response has been studied in several C57BL/6 murine models including DSS-induced colitis (22, 23), *Citrobacter rodentium*-induced intestinal inflammation [(24), *Clostridium difficile* infection (25), and food allergy (26)]. Even with the varying I3C doses used in these studies (ranging from 150–2,000 ppm), I3C supplementation activated AhR and reduced the associated immunopathology. In addition to direct effects on the intestinal immune response, supplementation of the diet with I3C also strongly shifts the gut microbiome composition (22, 23, 27). Although dietary I3C led to activation of AhR in each study, the specific changes in microbiota diversity varied between studies and occurred by both AhR-dependent and independent mechanisms. Thus, I3C alters gut health through two interacting pathways, by modulating the immune response and the microbiota.

Since both of these pathways can influence T1D progression (5, 28), in the present study we use the non-obese diabetic (NOD) mouse model of autoimmune diabetes to study the effects of dietary supplementation with I3C on gut immune-microbe interactions, and subsequent development of insulitis.

## Material and Methods

### Animals

NOD/ShiLtJ (NOD) mice were obtained from The Jackson Laboratory and maintained in the specific pathogen-free animal facility at Oregon State University. NOD.AhR-/- mice were generated by backcrossing B6.129-AHRtm1Bra/J onto the NOD/LtJ background for more than 13 generations. All experiments used female littermate-matched mice. All animal procedures were carried out following protocols approved by the Institutional Animal Care and Use Committee at Oregon State University.

### Preliminary Oral Gavage Studies

Cl-BBQ (11-chloro-7H-benzimidazo[2,1-a]benzo[de]Iso-quinolin-7-one; Chembridge) or I3C (Sigma) were dissolved in DMSO-Cremaphor-Peceol (30%:20%:50%) by sonicating in a 37 degree water bath for 1 h. Female NOD mice were administered 200 ul of Cl-BBQ (45 mg/kg), I3C (250 mg/kg) or the vehicle control by oral gavage. Mice were sacrificed at 6 and 24 h after oral gavage to measure AhR activation.

### Dietary I3C

NOD mice were fed normal chow until 7 weeks of age when they were transitioned to either a synthetic diet (AIN93M; Research Diets) alone or supplemented with 2,000 ppm I3C. Mice were maintained on these diets through the remainder of the study (12 weeks). Food consumption was tracked daily by weighing the remaining food for the first week of the study and then weekly from weeks 8 to 12. Body weight was likewise measured daily for the first week and then once a week for the remainder of the study. Two independent experiments containing three or four individual birth cohorts were conducted.

### Real-Time PCR and *Cyp1a1* Measurement

The liver and small intestine were removed from mice and stored in RNAlater for immediate stabilization prior to RNA isolation using RNeasy Mini Kit (Qiagen). cDNA was synthesized using the High-Capacity cDNA Reverse Transcription Kit (Applied Biosystems). qPCR reactions were performed on an Agilent Stratagene Real-Time PCR system (Applied Biosystems) using SYBR Green/ROX Master Mix from SA Biosciences. Cyp1a1 levels were normalized to Actb using primers from SA Biosciences.

### Assessment of Disease Progression

Insulitis was scored on sequential hematoxylin and eosin (H&E) stained pancreas sections separated by 200 μM with at least 50 islets scored per pancreas. Islets were scored as no infiltration, less than 50% infiltration or greater than 50% infiltration.

### Cell Isolation and Flow Cytometry

At 12 weeks of age, mice were sacrificed, pancreatic lymph node (PLN), spleen, and the small intestine were excised. Single cell suspensions were prepared from the spleen, PLN, and Peyer’s Patches by mechanical disruption between frosted slides. Splenoyctes were further processed by hypotonic red blood cell lysis. Isolation of lamina propria (LP) cells and intraepithelial lymphocytes (IEL) from the small intestine was performed as previously described (29). Briefly, Peyer’s patches were excised and the epithelial layer of the small intestine was isolated through sequential washes in a 5 mM EDTA and 0.145 mg/ml DTT solution while stirring at 37°C. LP cells were isolated from the remaining intestinal tissue by mincing and digesting the tissue using 0.2 U/ml Liberase TM and 0.05% DNase while shaking at 37°C. The digested tissue was washed three times with a 3% FBS solution and successively filtered after each wash through two 70 μm filters and one 40 μm filter in preparation for cell staining.

Cells were stained with fixable viability dye (eBioscience), Fc receptors were blocked with rat IgG (Jackson ImmunoResearch) and the cells were stained with the following antibodies CD45 (30-F11), CD4 (RM4-4), Nrp1 (3DS304M) from eBioscience, Lag3 (C9B7W), CD25 (PC61) and CD210 (1B1.3a), CD8 (53-6.7) from BD Biosciences and Tim3 from Biolegend. For intracellular staining, cells were fixed and permeabilized using the Foxp3 Fixation/Permeabilization buffer (eBioscience) and stained with Tbet (4B10) and Foxp3 (FJK168) from eBioscience, and RORγt (Q31-378) from BD Biosciences. For cytokine staining in splenoyctes, cells were stimulated with PMA, ionomycin, brefeldin A, and monensin (eBioscience) for 4 h in culture and stained with IFNγ (XMG1.2), IL-10 (JES5-16ES), IL-22 (1H8PWSR) from eBioscience and IL-17 (TC11-18H10) from BD Biosciences.

Data were acquired on a Cytoflex flow cytometer (Beckman Coulter). Data were compensated and analyzed using FlowJo (Treestar) software. Fluorescence minus one (FMO) controls were used for setting gates for analysis.

### 16S rRNA Gene Sequencing Analysis

For microbial measurements, fresh stool pellets were collected at 7, 8, and 12 weeks of age and immediately stored at -80°C. To extract microbial DNA, frozen fecal pellets were resuspended in 1.4 ml ASL buffer (Qiagen) and homogenized with 2.8 mm ceramic beads followed by 0.5 mm glass beads using an OMNI Bead Ruptor (OMNI International). DNA was extracted from the entire resulting suspension using QiaAmp mini stool kit (Qiagen) according to manufacturer’s protocol. DNA was quantified using Qubit broad range DNA assay (LifeTechnologies).

The primers 319F and 806R were used to amplify the V3-V4 domain of the 16S rRNA using a two-step PCR procedure. In step one of the amplification procedure, both forward and reverse primers contained an Illumina tag sequence (bold), a variable length spacer (no spacer, C, TC, or ATC for 319F; no spacer, G, TG, ATG for 806R) to increase diversity and improve the quality of the sequencing run, a linker sequence (italicized), and the 16S target sequence (underlined). In step two, each sample was barcoded with a unique forward and reverse barcode combination using forward primers (**AATGATACGGCGACCACCGAGATCTACAC***NNNNNNNN*TCGTCGGCAGCGTC) with an Illumina P5 adapter sequence (bold), a unique 8 nt barcode (N), a partial matching sequence of the forward adapter used in step one (underlined), and reverse primers (**CAAGCAGAAGACGGCATACGAGAT***NNNNNNNN*GTCTCGTGGGCTCGG) with an Illumina P7 adapter sequence (bold), unique 8 nt barcode (N), and a partial matching sequence of the reverse adapter used in step one (underlined). The final product was quantified on the Qubit instrument using the Qubit Broad Range DNA kit (Invitrogen) and individual amplicons were pooled in equal concentrations. The pooled library was cleaned utilizing Ampure XP beads (Beckman Coulter) then the band of interest was further subjected to isolation *via* gel electrophoresis on a 1.5% Blue Pippin HT gel (Sage Science). The library was quantified *via* qPCR followed by 300-bp paired-end sequencing using an Illumina MiSeq instrument in the Genome Center DNA Technologies Core, University of California, Davis. The QIIME 2 (30) bioinformatics pipeline (v. 2018.8.0) was used to demultiplex and quality filter the forward-end fastq files. Denoising was performed using DADA2 (31). The raw data can be accessed at NCBI Sequence Read Archive (SRA) (Accession #PRJNA679964).

### Data Pre-Processing

Host parameters were first normalized by birth cohort. Host parameters that were analyzed in both independent experiments were subsequently normalized across the two studies. For microbial data, after excluding amplicon sequence variant (ASV) singletons, a threshold of 99.5% cumulative abundance across all samples in both experiments was used to select the most abundant ASVs for downstream analysis. Following this, ASVs were relativized per million and quantile normalized (per experiment), then log2 transformed for downstream analysis.

### Reconstruction of Transkingdom Network

The network analysis has been performed as described in previous papers (32, 33), except for the minor modifications outlined below. Spearman rank correlations were conducted for all experimental groups (Control and I3C for each of the two experiments) between all pairs of ASVs and host parameters. Edges that did not demonstrate the same sign of correlation direction (positive or negative) across all groups and those that were not compliant with causality principles (34) were removed. Similarly, those edges that did not contain nodes consistent in fold change direction across experiments were also removed. The metacor function of the meta package [v 4.12-0 (35);] in R (v 3.5.1) was used to calculate the fixed effect model p-values based on Fisher’s Z-Transformation of proportions of the correlations across the four groups. Prior to calculating FDRs, the correlations between microbiota and phenotypes were first filtered for those that had an individual p-value <0.6 across all groups, with the exception of the PP_RORgt+Foxp3-<==>ASV21 and IEL_RORgt+Foxp3-<==>ASV290 edges (FDR = 0.135 for both). These two additional edges were kept in the network despite not passing individual p-value thresholds due to the high biological importance of the RORγt^+^Foxp3^-^ cell populations. Therefore, we checked for edges connecting to that cell population that barely missed making the network. FDR was then calculated on the correlation p-values for each of the following groups: (1) between microbes; (2) between microbes and host parameters; (3) between host parameters. Microbe-host parameter edges with an FDR < 0.15 were retained in the network. Edges between microbiota were required to have an FDR < 0.05 while those between host parameters were required to have an FDR <0.1.

### Network Analysis

Networks were visualized using Cytoscape v3.7.2 (36). Silva IDs were used in labeling the important microbes (as determined by their high normalized bipartite betweenness centrality [BiBC] and degree). The Python module NetworkX v2.2 was used to calculate BiBC and degree between groups, as well as to randomly generate the 10,000 networks used in validating the BiBC and degree results. BiBC values were calculated as previously described (32), then normalized by the number of nodes in each group. The 10,000 binomial random (Erdos-Renyi) networks were generated from the G(m,n) ensemble with m = 221 (to match the number of edges in the real network) and n = 109 (the number of nodes). The 2D contour histogram BiBC-degree distribution was plotted using the online tool Plotly (https://plot.ly/). Probability density was used as a measurement of the likelihood of randomly finding a node with the given BiBC and degree (or higher). A large value (i.e. a dark-colored space in the contour map) indicates that a node in that area typically occurs in random networks size-matched to the network generated from the data.

### Statistical Analysis

With the exception of the network analyses, statistical analyses were performed using Graphpad Prism. For insulitis and host parameters, data were normalized by birth cohort/littermates. For comparing two groups, a Student’s t test was performed. For multiple comparisons, one-way ANOVA with Tukey’s test was used. P <0.05 was considered statistically significant. All plotted data points represent an individual mouse.

## Results

### Dietary I3C Strongly Activates AhR in the Intestine but Induces Limited Systemic AhR Activation

Activation of the AhR by I3C supplementation has not been previously studied in NOD mice. To determine the extent by which I3C can induce AhR activation, we first measured activation following oral gavage with I3C as compared to Cl-BBQ, a high affinity AhR ligand previously shown to suppress T1D in NOD mice (10, 12). In C57BL/6 mice, 250 mg/kg of I3C (or dietary equivalent) is on the higher range of previously reported doses used to activate the AhR (22–24, 26, 27, 37) [; higher doses (> 500 mg/kg) of I3C are associated with neurotoxicity and increased mortality (20)]. To account for the AhR^d^ allele, which has a ~10 fold lower sensitivity to AhR ligands (12, 38), a dose 250 mg/kg I3C was chosen to maximize AhR activation and minimize toxicity.

**Figure 1:**
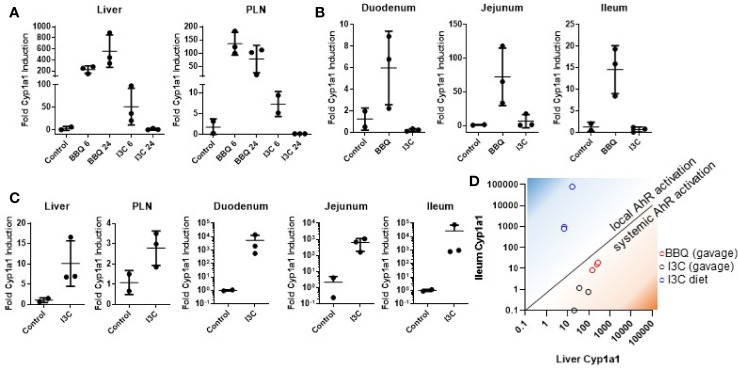
Dietary I3C strongly activates AhR in the intestine but not systemically. *Cyp1a1* expression was measured in the liver and pancreatic lymph node of NOD mice following treatment with either I3C (250 mg/kg) or Cl-BBQ (45 mg/kg) at 6 and 24 h post-oral gavage **(A)** and in the small intestine 6 h post-oral gavage **(B)**. **(C)** *Cyp1a1* was analyzed after one week of dietary I3C (2,000 ppm) in the indicated organs. **(D)** Comparison between local (ileum) and systemic (liver) AhR activation by Cl-BBQ (gavage), I3C (gavage), and dietary I3C.

NOD mice were administered I3C by oral gavage and *Cyp1a1* induction was measured in the liver, pancreatic lymph node, and small intestine (duodenum, jejunum, and ileum). AhR response elements upstream of *Cyp1a1* make it a highly sensitive target of AhR and, as a result, the induction of *Cyp1a1* is a commonly used biomarker for AhR activation (39). The positive control, Cl-BBQ, resulted in sustained *Cyp1a1* induction in the liver and pancreatic lymph nodes at 6 and 24 h post-oral gavage. In contrast, I3C only resulted in transient *Cyp1a1* induction, which was 4- and 20-fold lower in the liver and pancreatic lymph node, respectively, at 6 h post-oral gavage. By 24 h after I3C administration, *Cyp1a1* was back to baseline (**Figure 1A**). Furthermore, I3C did not induce *Cyp1a1* in the duodenum, jejunum, and ileum of the small intestine following oral gavage (**Figure 1B**).

In contrast to gavage administration, one week of diet supplementation with I3C (2,000 ppm, equivalent to 250 mg/kg/day based on food consumption) was capable of inducing strong *Cyp1a1* in the small intestine (median of ~1,000-fold increase; **Figure 1C**). However, as with oral gavage, AhR activation was likewise limited systemically following dietary exposure. Given the apparent lack of systemic AhR activation following dietary I3C supplementation, it was difficult to predict how it would impact T1D pathogenesis. However, the ability of I3C to induce *Cyp1a1* locally in the intestine (**Figure 1D**), provided an unexpected opportunity to determine how AhR activation in the gut alters the development of T1D.

### Dietary I3C Exacerbates Insulitis in Nonobese Diabetic Mice

**Figure 2:**
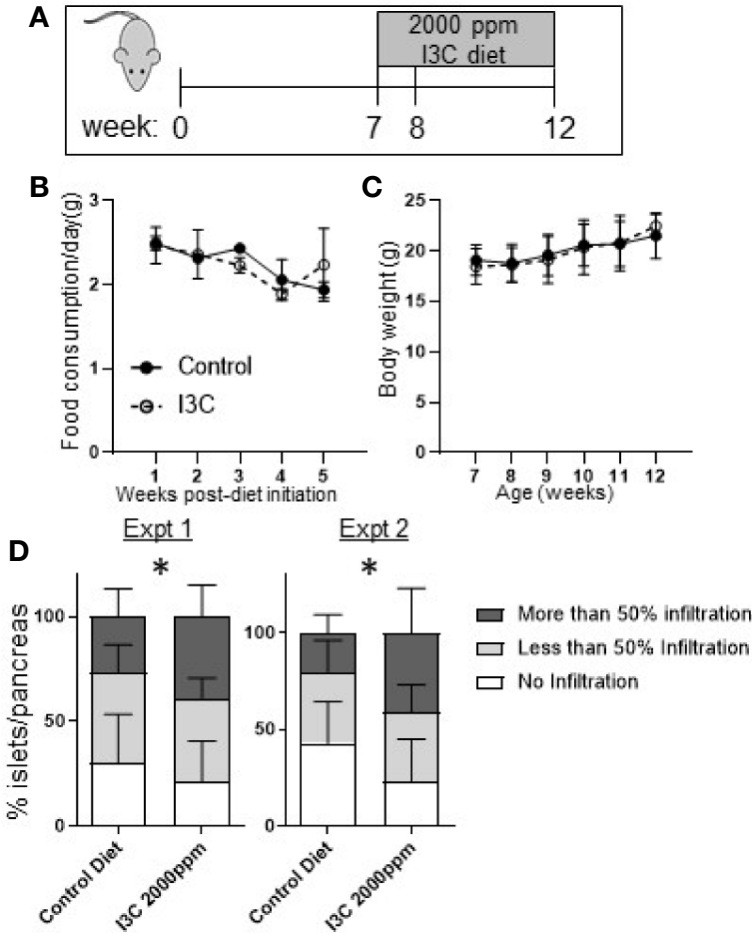
Dietary I3C exacerbates insulitis in NOD mice. **(A)** Schematic of experimental design. From 7 to 12 weeks of age, NOD mice were maintained on a synthetic AhR ligand-free diet or diet supplemented with 2,000 ppm I3C. Stool samples were collected at 7, 8, and 12 weeks of age (at the time of sacrifice). Food consumption **(B)** and body weight **(C)** were measured weekly. **(D)** In two independent experiments, insulitis was scored as no infiltration, less than 50%, and greater than 50% of islets infiltrated. Data are presented as the average from each mouse per group. Experiment 1: n = 9 mice/group; Experiment 2: n = 7–8 mice/group. *p < 0.05.

To determine if strong AhR activation in the intestine was sufficient to suppress insulitis, NOD mice were fed a diet supplemented with 2,000 ppm I3C from seven to twelve weeks of age, during which time insulitis is known to progress. Initiating the dietary regimen at this timepont was selected to correspond to the treatment timing in our previous studies with Cl-BBQ and TCDD (10). At seven weeks of age, NOD mice were randomized by litter, cage, and weight then assigned to a control or I3C-diet fed group (**Figure 2A**). Mice fed an I3C-supplemented diet showed no difference in food consumption or body weight compared to control mice (**Figures 2B**, **C**). Mice averaged 2–2.5 g of food/week resulting in an average exposure of 190–260 mg/kg/day of I3C.

At 12 weeks of age, mice were euthanized and islet infiltration was scored in the sectioned pancreas. Unexpectedly, mice given the diet supplemented with I3C had significantly increased insulitis. The increase in insulitis was primarily a reflection of the significant increase in the percentage of islets per pancreas that were heavily infiltrated (50% infiltration, p < 0.05) in mice fed the I3C-supplemented diet; the percentage of islets with <50% infiltration was unchanged between treatment groups. This pattern was observed in two independent experiments (**Figure 2D**).

### Dietary I3C Increases Th17 Cells in the Intestine

**Figure 3:**
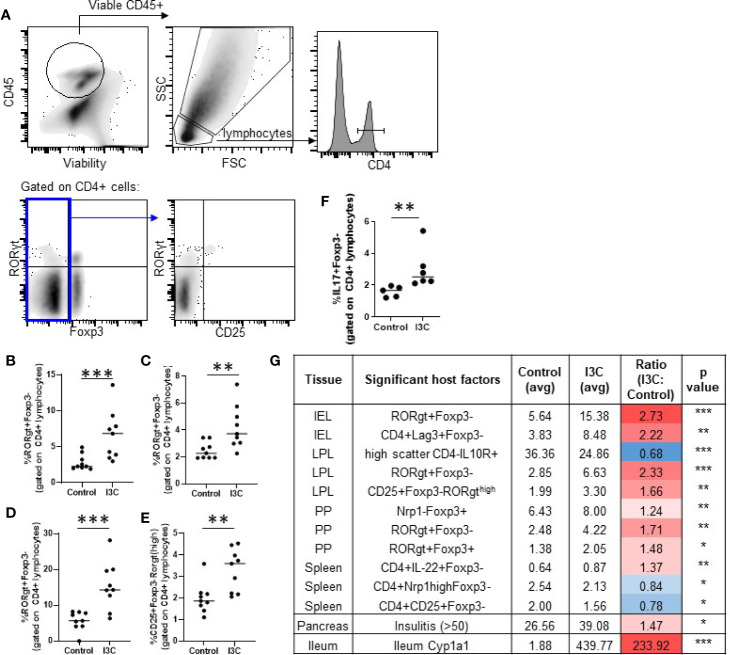
Dietary I3C increases Th17 cells in the intestine. Representative gating strategy **(A)** and population frequency of Th17 cells in the small intestine lamina propria **(B, E)**, Peyer’s patches **(C)**, and intraepithelial layer **(D)**. A subset of lamina propria cells were stimulated with PMA/ionomycin and stained for IL-17 **(F)**. Table of significant changes in all analyzed populations **(G)**. *p < 0.05; **p < 0.01; ***p < 0.001.

Th17 cells and Tregs (Foxp3^+^ and Tr1) are targets of AhR activation and implicated in promoting and inhibiting the autoimmune destruction of beta cells, respectively (16, 18, 19, 40–42). Therefore, alterations in markers representing CD4^+^ T cell helper subsets were examined in the spleen, pancreatic lymph node, Peyer’s patches, the small intestine lamina propria, and the small intestine intraepithelial cells. Significant increases were found in CD4^+^Foxp3^-^RORγt^+^ Th17 cells in the lamina propria (2.3-fold increase), intraepithelial layer (2.7-fold increase), and Peyer’s Patches (1.6-fold increase; **Figures 3A–D**). A subset of lamina propria CD4^+^Foxp3^-^ cells expressing high levels of RORγt expressed the T cell activation marker CD25^+^ and were significantly increased as well (1.7-fold increase; **Figure 3E**). Confirming that dietary I3C increased Th17 cells, LPL cells from a subset of mice were stimulated with PMA/ionomycin and IL-17 was measured by flow cytometry (**Figure 3F**). No significant changes in Tr1 cells, Th1 cells, and Th17 cells were found in the spleen or pancreatic lymph nodes, although a trend toward increased Th17 cells was observed in the PLN (**Supplementary Figure 1**), consistent with the minimal AhR activation at these sites. While the majority of significant changes in immune cell markers were identified in the intestine, some minor yet significant populations were changed in the spleen. Significant changes in all analyzed populations are listed in **Figure 3G**.

### Dietary I3C Alters the Gut Microbiome

Since modulation of immune cell populations in the gut by dietary I3C can arise from AhR-mediated changes in gut microbiota diversity, we examined whether dietary I3C altered gut microbial composition in NOD mice. Stool samples were collected prior to the dietary intervention at 7 weeks of age, after one week of the diet, and at the time of sacrifice at 12 weeks of age.

**Figure 4:**
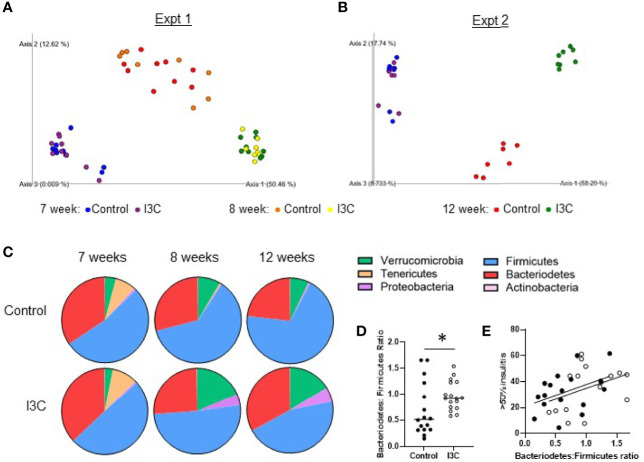
Dietary I3C alters the gut microbial community. **(A, B)** Principle coordinate analysis of microbiome composition of stool samples collected prior to the dietary intervention at 7 weeks of age, after one week of the diet (8 weeks, experiment 1 only), and at the time of sacrifice at 12 weeks of age. **(C)** Phylum composition in mice fed a control or I3C-supplemented diet. **(D)** Ratio of Bacteriodetes to Firmicutes. **(E)** Correlation between severe insulitis and Bacteriodetes to Firmicutes ratio in control-diet (solid circles) and I3C-diet (open circles) fed mice. *p < 0.05.

Within one week of starting the new diets, there was a strong shift in the microbial communities of both groups of mice fed the synthetic control diet or the I3C-supplemented diet (**Figures 4A, B**). Samples from mice in the control diet group and, to a greater extent, the I3C-supplemented diet group separated from the 7 week samples on the PC1 axis, which accounted for 50–58% of diversity in two independent experiments. Samples collected from the control diet group separated from the I3C-supplemented group on the PC2 axis, which accounted for 12–17% of diversity. Interestingly, changes in microbial diversity were established within the first week of the synthetic diet (**Figure 4A**).

At the phylum level (**Figure 4C**), the synthetic diet, regardless of supplementation, reduced the abundance of Tenericutes and increased Verrucomicrobia. The samples from mice fed the I3C-supplemented diet diverged from the control-diet group with an increase in Proteobacteria and Verrucomicrobia. In both NOD mice and patients with T1D, an increased ratio of Bacteriodetes to Firmicutes is implicated in the development of T1D (5). Consistent with dietary I3C increasing insulitis, there was a significant increase in the ratio of Bacteriodetes : Firmicutes at 12 weeks of age in mice fed the I3C supplemented diet (**Figure 4D**). This corresponded with a positive trend (p = 0.06) between the Bacteriodetes : Firmicutes ratio and percentage of islets with greater than 50% infiltration (**Figure 4E**).

### Transkingdom Network Analysis Identifies Bacteria in the Genera *Intestinimonas*, *Ruminoclostridium*, and Unclassified Lachnospiraceae as Key Responders to Dietary I3C

**Figure 5:**
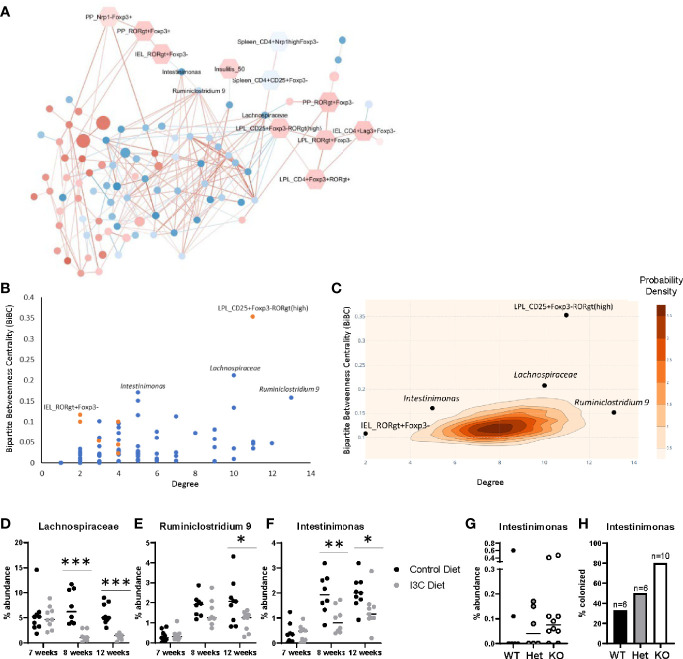
Transkingdom network analysis identifies key host-microbe interactions. **(A)** Correlation network of ASVs (circles) and phenotypes (hexagons). Node color indicates the average median fold change (red is increased, blue is decreased) across all experiments. ASV node size indicates the average abundance of the ASV across both experiments. Edge color represents the average spearman correlation coefficient (red is positive, blue is negative) across all experimental groups. **(B)** BiBC-degree distribution of all nodes in the network, the highest of which are marked. Blue are ASVs and orange are phenotypes. **(C)** 2D-contour histogram of the nodes with highest BiBC and degree from 10,000 randomly generated networks. Darker areas indicate higher probabilities of randomly finding a node with that degree and BiBC. **(D–F)** Temporal abundance of Lachnospiraceae, *Ruminiclostridium* 9, and *Intestinimonas* at 7, 8, and 12 weeks of age after treatment of I3C. **(G)** Abundance of *Intestinimonas* in NOD AhR knockout mice at 12 weeks of age. **(H)** Percentage of mice colonized with *Intestinimonas*. *p < 0.05; **p < 0.01; ***p < 0.001.

To begin to identify which ASVs may be implicated in the immunological changes that arose following dietary I3C, we constructed a transkingdom network. Transkingdom networks have been successfully used to tease out causal interactions between mammalian host phenotypes and their colonizing microbiota (43). Here, the transkingdom network (**Figure 5A**) was constructed by computing correlations between host factors (hexagons) that significantly changed in mice fed the I3C-supplemented diet (**Figure 3G**) and ASVs identified at 12 weeks of age (circles). The network retained 108 nodes (red: increased and blue: decreased in I3C treated mice) and 221 edges, of which there were 179 positive correlations (red lines) and 42 negative correlations (blue lines).

The network was then interrogated to predict which host factors and microbes were most likely to play a role in the response to dietary I3C. We calculated two properties of the nodes, degree and bipartite betweenness centrality (BiBC) (**Figure 5B**); degree is the number of nodes each individual node interacts with, and measures the direct impact of one node has on other parameters in the system; bipartite betweenness centrality calculates the number of times the node lies in the shortest path connecting two groups of nodes (host parameters and ASVs). A node with a high BiBC acts as a bottleneck and thus is a regulator between processes of the system. Collectively nodes with high degrees and centrality are predicted to be critical in defining host-microbe interactions that are involved in the response to I3C. By plotting bipartite betweenness centrality and degree, we identified three ASVs, one with a high BiBC (*Intestinimonas*) and two with both high BiBC and high degree (*Ruminiclostridium* 9 and unclassified Lachnospiraceae) which likely regulate the abundance of other gut microbes. *Intestinimonas*, *Ruminiclostridium*, and Lachnospiraceae were decreased in I3C treated mice and are members of phylum Firmicutes. On the host side, two parameters showed high BiBC, intraepithelial CD4^+^RORγt^+^Foxp3^-^ cells (**Figure 3D**) and CD4^+^CD25^+^Foxp3-RORγt(high) lamina propria cells (**Figure 3E**) and therefore likely influence or are influenced by microbial interactions.

The statistical importance of these five nodes was further validated by randomly generating 10,000 networks and comparing the degree and BiBC of these specific nodes to those in a randomly generated model. Indeed, the degrees and BiBC of the five nodes had a low probability of occurring randomly (**Figure 5C**).

While *Ruminiclostridium* 9, *Intestinimonas*, and Lachnospiraceae likely play a role in host interactions, it is unclear if their change in abundance was due to, or resulted in, changes in the intestinal immune response after I3C treatment. Therefore, we looked at the temporal abundance of these microbes (**Figures 5D–F**). Within one week of switching from the normal chow diet to the synthetic diet there was an increase in abundance in both *Ruminiclostridium* 9 and *Intestinimonas* regardless of I3C supplementation. However, the increase in abundance of *Intestinimonas* was larger in the control group in comparison to the I3C-diet group, and this pattern remained through 12 weeks of age. In contrast, the comparatively lower abundance of *Ruminiclostridium* 9 was not significant until 12 weeks of age. The transition from the normal diet to the synthetic diet did not significantly alter the abundance of the unclassified Lachnospiraceae ASV, however supplementation with I3C depleted the abundance of this family at both 8 and 12 weeks of age. The immediate differential abundance in *Intestinimonas* and Lachnospiraceae may reflect a primary response to AhR-mediated changes in the gut immune system, whereas the delayed reduced abundance in *Ruminiclostridium* 9 may be secondary to changes in microbial composition.

Since I3C could alter colonization with Firmicutes directly or indirectly of AhR intestinal activation, we verified if the abundance of these bacteria were affected by genetic manipulation of AhR expression. To ensure relevance of this experimental group for effect on diabetes, we used NOD AhR knockout mice at 12 weeks of age when approximately 60% of islets are infiltrated, but prior to clinical manifestations. Among the three above mentioned microbes, only *Intestinimonas* showed lower abundance with gene dosage. Accordingly, gut colonization by the only member of the *Intestinimonas* genus detected in these mice, was found in 33% of wildtype mice and 50% of heterozygous mice were colonized with *Intestinimonas*, 80% of their knockout littermates were colonized (**Figures 5G, H**). Taken together, *Intestinimonas* abundance is negatively regulated by AhR and therefore the decrease in this genus is likely to be an effect of I3C-mediated AhR signaling in the intestine.

## Discussion

As a molecular switch between opposing immune responses (regulatory and inflammatory), activation of the AhR can either protect from or promote the development of immune-mediated diseases. Understanding how immune modulation is impacted by different AhR ligands, their routes of administration, and their biodistribution is important for determining if AhR ligands may act as an environmental risk factor for T1D development, and/or how AhR can be exploited as a target for T1D treatment. In the current study we found that gut-localized AhR activation by I3C increased Th17 cells and exacerbated T1D in NOD mice, in contrast to our previous findings that systemic AhR activation by Cl-BBQ and TCDD reduced Th17 cells and prevented the development of T1D (10, 44). These results suggest that the site of AhR activation influences the outcome of CD4^+^ T cell differentiation.

These contrasting effects on CD4^+^ T cell differentiation do not appear to be a consequence of the extent of AhR activation. Using an alloresponse model, we previously found that if you can normalize the extent AhR activation, CD4^+^ T cell differentiation follows a predictable pattern. Low levels of AhR activation promote Th17 cells and high levels promote Tr1 cell differentiation (15). While dietary I3C only led to limited AhR activation in the periphery in comparison to Cl-BBQ, the opposite occurred in the small intestine; dietary I3C induced ~2,000-fold higher induction of *Cyp1a1* than Cl-BBQ in the ileum. By strongly activating *Cyp1a1* in the small intestine, it is possible that the AhR activating catabolites of I3C (e.g. ICZ), were rapidly metabolized, inducing feedback control that prevented systemic distribution (45). Despite inducing an environment where I3C led to strong local AhR activation, Th17 cells were increased in the intestine. The organ site of AhR activation (intestine vs periphery) with different microenvironments, cell composition, and architecture may be a missing variable controlling the outcome of AhR-signaling in CD4^+^ T cells.

While systemic Th17 cells are implicated in the development of T1D (17–19, 41), it is less clear how intestinal Th17 cells impact disease development. Their role during T1D is further complicated by studies in which intestinal Th17 are ascribed both proinflammatory and homeostatic properties. These differing functionalities may be dependent on how intestinal Th17 cells develop in concert with the microbiome. Th17 cells appear in mice at the time of wean corresponding to cecum development and establishment of the microbiota (46). It is conceivable, therefore, that initiating dietary I3C at the time of wean, rather than at 7 weeks of age, could negate the I3C-mediated increase in Th17 cells. One study in germ-free C57BL/6 mice, showed that Th17 cells do not develop in the small intestine lamina propria, and fecal transfer from SPF mice restored IL-17 production. In contrast, a study found that germ-free NOD mice have both increased Th17 cells and Th1 cells resulting in exacerbated insulitis (47). By comparing diabetes incidence in multiple housing facilities, it was found that colonization with segmented-filamentous bacteria (SFB) could be used to stratify an increase in Th17 cells and reduced T1D incidence (48). The protective effect of SFB requires a diverse microbiota, as monocolonization with SFB does not protect from T1D (49). Of note, AhR regulates SFB colonization (50), although SFB was not present in our mouse colony during the current study. A recent paper by Omenetti et al. poses that these contradictory findings are due to the presence of two different types of resident Th17 cells, homeostatic (which can be generated from SFB) and inflammatory (which can be generated from *C. rodentium*) (51). The homeostatic Th17 cells play a role in barrier protection and have a limited metabolic program whereas the inflammatory Th17 cells produce IFNγ and are able to migrate into the periphery. Therefore, the functionality of Th17 cells depend on their interaction with specific microbes, and may explain the conflicting literature on gut Th17 cells and T1D pathogenesis.

Intestinal Th17 cell differentiation following AhR activation may proceed through both direct CD4^+^ T cell signaling and indirectly through AhR-mediated modulation of the gut microbiome. AhR is highly expressed in Th17 cells. Under Th17 differentiating conditions AhR activation, in combination with Stat3, activate Aiolos, inhibiting IL-2, and promoting Th17 differentiation (52). Th17 differentiating conditions are present in the intestine originating from microbial metabolites (including AhR ligands), microbe-mediated PRR activation, and microbial-secreted ATP, where binding to purinergic receptors expressed on LP dendritic cells along with TLR activation increases Th17-skewing IL-6 and TGF- β production (53, 54) Microbial activation of the NLRP3/IL-1β signaling pathway can also induce intestinal Th17 polarization (55), although, interestingly, AhR signaling has been shown to negatively regulate inflammasome activation (56). Thus, it likely that a combination of (and sometimes opposing) Th17-promoting factors could be implicated in the dietary I3C-mediated increase in intestinal CD4^+^RORγt^+^Foxp3^-^ cells in NOD mice.

In our study, dietary I3C strongly skewed the gut microbial community and increased the Bacteriodetes : Firmicutes ratio. In mouse studies, this increased ratio is positively correlated with T1D, and prediabetic children have an increased abundance of Bacteriodetes (5, 57, 58). However, the utility of using this ratio as predictive for T1D development is inconsistent, and is further complicated by the site of bacteria collected for analysis. Fecal bacteria (as measured in this study), have higher abundance of Bacteriodetes compared to cecal content (59). Using a transkingdom network analysis, we identified three genera belonging to the Firmicutes phylum that are predicted to be involved in the immune modulation by dietary I3C, *Intestinimonas, Ruminiclostrdium 9* and unclassified Lachnospiraceae. Based on their network properties and follow up studies in AhR knockout mice, we predict that *Intestinimonas* is directly regulated by AhR activation, whereas *Ruminiclostridium 9* and Lachnospiraceae may be altered indirectly in response to AhR-host-microbe interactions. All three of the bacteria identified as key contributors to the network are butyrate producers (60–62); butyrate helps promote intestinal integrity and increases Tregs. NOD mice fed a diet that increases butyrate production by the gut microbiota had decreased insulitis (63). Butyrate-mediated protection from T1D corresponded with tolerized DCs and an increase in Tregs in the colon but not in the pancreatic lymph node. The protective role of butyrate is consistent with the reduction in butyrate-producing bacteria in mice given dietary I3C. Interestingly, a recent study showed that i.p. injection of I3C resulted in an increase in butyrate-producing gut bacteria in a C57BL/6 mouse model of TNBS-induced colitis (37). I3C-mediated resolution of disease and increase in butyrate-producing microbes were dependent on the induction of IL-22. IL-22 has been reported as one of the main mechanisms by which AhR can regulate the gut microbiome and immune homeostasis (37, 50, 64–66). However, in our study with NOD mice, I3C did not alter IL-22 expression in the small intestine when measured by qPCR or by flow cytometry (data not shown).

Additional differences between the AhR response in NOD and C57BL/6 mice are highlighted when comparing our study with findings in a model of oral tolerance. In NOD mice, TCDD and Cl-BBQ (when administered at a dose-rate to match the activation of AhR by TCDD) prevent insulitis (10), and I3C exacerbates disease. Conversely, in the oral tolerance model, I3C promotes oral tolerance and TCDD breaks oral tolerance (26, 67). Interestingly, in these C57BL/6 mice, TCDD induced an order of magnitude higher *Cyp1a1* in the small intestine compared to I3C, and led to a small, but significant increase in Th17 cells in the mesenteric lymph nodes. Thus, an important unresolved question is why does I3C lead to immune regulation, expand intestinal Tregs and promote IL-22 production in other C57BL/6 mouse models, when the opposite occurs in NOD mice. One clue might come from a study that looked at the impact of AhR allele on the ability of dietary broccoli to suppress colitis. Using C57BL/6 mice that express either the AhR^b^ allele (wildtype, high affinity) or congenic C57BL/6 mice with the reduced sensitivity AhR^d^ allele (68), it was found that AhR sensitivity determined disease outcome. In the wild type C57BL/6 mice, dietary broccoli attenuated colitis and reduced Th17 cells, similar to the effect of I3C in colitis models. In contrast, AhR^d^ mice fed the broccoli-supplemented diet had a 1.6-fold increase in splenic Th17 cells. Since NOD mice express the lower affinity AhR^d^ allele, (12), this genetic difference may represent an additional factor controlling AhR-mediated CD4^+^ T cell modulation. The differences in allele sensitivity may also explain contradictory findings in regard to IL-22. In NOD mice, pancreatic islets have defective *Il22* expression compared to BALB/c (AhR^b^) mice despite having similar concentrations of AhR ligands in their feces and serum (69). Humans express an AhR with similar ligand sensitivity to the murine AhR^d^ allele, thus the AhR^d^ model may be more appropriate for predicting immune modulation in humans in response to dietary AhR ligands.

The restriction of AhR activation to the intestine following dietary I3C and the promotion of insulitis was unexpected. However, these results provided new insights on the role of AhR allele sensitivity, intestinal Th17 cells and gut microbial composition during the development of T1D. Additionally, these findings highlight potential risks associated with dietary I3C supplementation.

## Data Availability Statement

The data presented in the study are deposited in the NCBI Sequence Read Archive, accession number PRJNA679964.

## Ethics Statement

The animal study was reviewed and approved by the Institutional Animal Care and Use Committee at Oregon State University.

## Author Contributions

HK, NN, JP, and AE performed the experiments. NS, AM, and AE contributed to the conception and design of the study. NM, AM, and AE analyzed the data. AE, NK, SK, and NS provided funding for the project. AE, NN, and NK wrote the manuscript. All authors contributed to the article and approved the submitted version.

## Funding

This work was supported by the NIDDK (grant numbers 1K99DK117509-01 and 4 R00 DK117509-03 awarded to AE, R01 DK103761 awarded to NS) and NIEHS (grant number 5R01ES016651 awarded to NK and SK) and startup resources from UC Davis.

## Conflict of Interest

The authors declare that the research was conducted in the absence of any commercial or financial relationships that could be construed as a potential conflict of interest.
