# Supplementary figures and images for: Dietary Indole-3-Carbinol Activates AhR in the Gut, Alters Th17-Microbe Interactions, and Exacerbates Insulitis in NOD Mice
Source: Front Immunol. 2021 Jan 21;11:606441. doi: 10.3389/fimmu.2020.606441 (PMC7858653; doi:10.3389/fimmu.2020.606441)

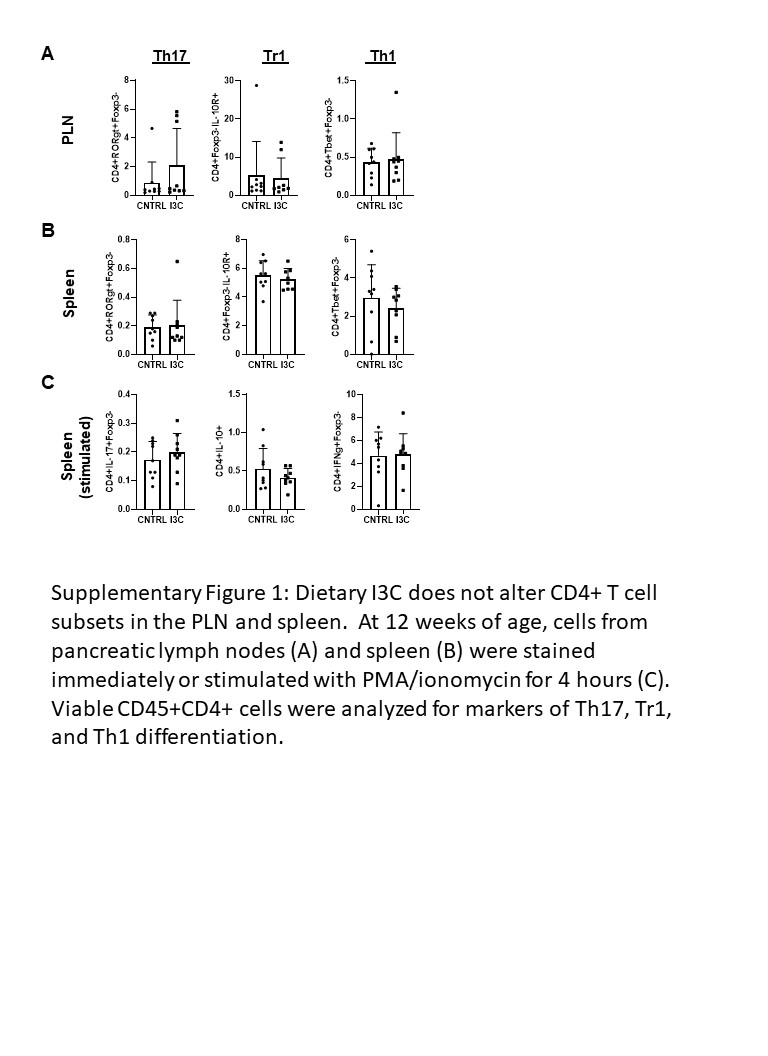

Supplement: Supplementary file 1 [file Image_1.jpeg]
